# Exploring the Influence of an E-Learning Sensory Processing-Based Module for Graduate Level Occupational Therapy Students on Clinical Reasoning: A Pilot Study

**DOI:** 10.1155/2017/6515084

**Published:** 2017-01-10

**Authors:** Bryan M. Gee, Jane Strickland, Kelly Thompson, Lucy Jane Miller

**Affiliations:** ^1^Idaho State University, Pocatello, ID, USA; ^2^STAR Institute for Sensory Processing Disorder, Greenwood Village, CO, USA; ^3^Department of Pediatrics, University of Colorado, Denver, CO, USA

## Abstract

The purpose of this study was to explore the effectiveness of a series of online, module-based instructional reusable learning objects (RLOs) targeted at entry-level, 1st year, Master of Occupational Therapy students. The content of the RLOs addressed knowledge and implementation of A SECRET, a parental reasoning approach for children with a sensory processing disorder, specifically sensory over responsiveness. Nine RLOs were developed and embedded within a commonly used learning management system. Participants (*n* = 8) were evaluated regarding their ability to discriminate between appropriate and inappropriate A SECRET strategies using a selected-response assessment. The participants' overall average score was 68%, a positive finding given the novelty of the instruction, assessment, and the content.

## 1. Introduction

Occupational therapy (OT) educators train entry-level OT students to identify, evaluate, and treat children with sensory processing difficulties and disorders [1]. This charge originates from the American Occupational Therapy Association's (AOTA) 2011 educational standards for Accreditation Council of Occupational Therapy Education (ACOTE) and AOTA's Blue Print for the Future of Entry Level Education [2]. According to both AOTA's accreditation standards and ACOTE's blueprint, entry level occupational therapists should demonstrate competency in assessing sensory needs and providing stimulation and environmental self-management strategies to those with sensory processing deficits [3].

It has been reported that 83–90% of occupational therapists working with pediatric populations provide interventions to address their clients' sensory processing deficits [4]. Green et al. [5] reported that a sensory processing approach was the third most frequent strategy in intervention with children with ASD. Furthermore, ACOTE requires students to “demonstrate an understanding of health literacy and the ability to educate and train the client, caregiver, family and significant others, and communities to facilitate skills in areas of occupation as well as prevention, health maintenance, health promotion, and safety” [3, p. 25] and “apply the principles of the teaching–learning process using educational methods to design experiences to address the needs of the client, family, significant others, colleagues, other health providers, and the public.” (p. 25).

Based upon the literature, it is evident that a wide array of children demonstrate sensory processing disturbances and that occupational therapists incorporate sensory processing approaches to address those difficulties [6–8]. The type of sensory processing frame of reference (e.g., Dunn's Model of Sensory Processing, Ayres Sensory Integration Intervention®, Nosology of Sensory Processing), type of comprehensive intervention strategy (e.g., Ayres Sensory Integration Intervention, Sensory Treatment and Research Center Sensory Processing Approach), specialized sensory based intervention (e.g., sound-based interventions, therapy ball chairs, weighted vests, deep pressure vests, Wilbarger Deep Pressure Brushing Protocol, and the Astronaut Training Program), and sensory motor intervention (e.g., therapeutic horseback riding, therapy balls) are taught in entry-level occupational therapy programs [1]. However, teaching a clinical reasoning process (e.g., A SECRET) to entry-level occupational therapy students as part of their defined curriculum has not been widely reported in the literature.

Occupational therapy has been employing diverse instructional technologies from hybrid (online/face-to-face) courses to exclusively online offerings for several years [9]. Furthermore, other health professions have used instructional technology and distributed learning as part of their entry-level programs, including pharmacy, nursing, physician assistant, speech language pathology, and physical therapy [10–12].

Interestingly, nursing, pharmacy, and physician assistant programs in the United Kingdom have been using learning objects (LOs) and/or reusable learning objects (RLOs) to bridge knowledge gaps due to content/course removal or to enhance skills and abilities without additional class time [11, 12]. Currently, however, there is limited information regarding the use of RLOs in occupational therapy entry-level education as mechanisms to enhance face-to-face or hybrid instruction with the exception of Gee et al. [13] who reported favorable opinions among occupational therapy students who used an RLO related to goal writing for existing and future coursework/clinical rotations.

In general, RLOs are “any digital resource that can be used and reused to support learning” [14, p. 6]. Reusable learning objects typically are small, discrete, self-contained digital objects that may be sequenced, combined, and used within a variety of instructional activities [14] including integration into formal lectures or as stand-alone elements for remediation or background knowledge development [11, 15]. Much like the educational handouts, manipulatives, and “objects” classroom teachers have always created and then shared with learners, RLOs afford even greater transportability beyond the confines of place and time.

Though RLOs within some of the health science education or professional programs [11, 12], there is a lack of published literature documenting the implementation of RLOs into the broader rehabilitation sciences for entry-level professional education, especially in occupational therapy. There are several characteristics distinguishing RLOs from other pedagogical tools and resources, including reusability, accessibility, interoperability, durability, granularity, sequencing, framing, stringing, and combinability.

**Figure 1:**
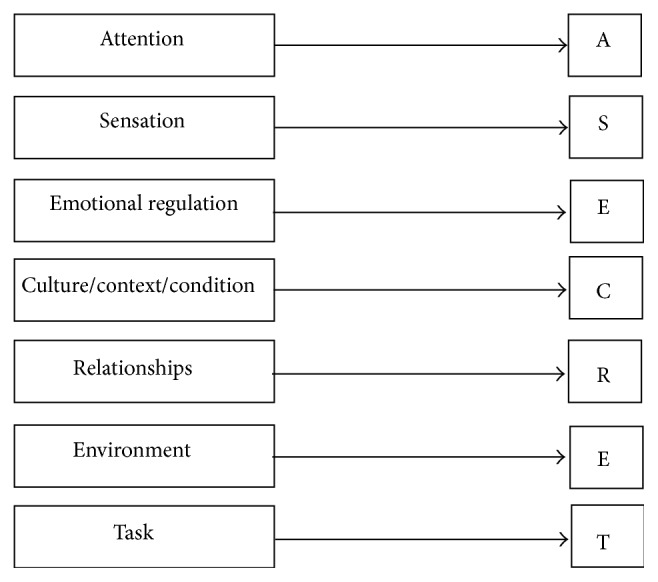
A SECRET framework.

The RLOs that were designed and developed related to A SECRET in this study intentionally employed these characteristics to increase the likelihood they would be instructionally sound. A SECRET is a mnemonic for (A) Attention, (S) Sensation, (E) Emotional Regulation, (C) Culture/Context/Condition, (R) Relationships, (E) Environment, and (T) Task [16] (see Figure 1). A SECRET [16, 17] is a problem-solving framework developed for parents and caregivers to enhance problem-solving abilities for their child/client's challenging sensory related behaviors. The framework attempts to capture how clinicians think about sensory related behaviors and the questions to ask that may lead to the design and implementation of strategies to reduce duration, frequency, and/or intensity of episodes for a child with SPD (sensory processing disorder) [16, 17].

The A SECRET framework is targeted to address sensory related behaviors among children who are diagnosed with SPD or have sensory processing difficulties that may comorbidly occur with other medical, behavioral, or developmental conditions [16, 17]. The A SECRET approach is grounded in the theoretical Ecological Model of Sensory Modulation [18]. The purpose of the study was to track student performance on a selected response, case based scenario after completing instructional modules and RLOs related to sensory processing and A SECRET.

## 2. Method

### 2.1. Participants

The participants of this study were eight MOT students between the ages of 20 and 50 years, who were in the first 12 months of the program (a three-year Master of Occupational Therapy degree) at an intermountain west public university. All the participants met the minimum criteria for admission into the MOT program and have a bachelor's degree in an associated area (University Studies, Psychology, Sociology, Exercise Science, etc.).

### 2.2. Inclusion/Exclusion Criteria

Individuals who took part met the following inclusion criteria. Specifically, they were 1st year occupational therapy students who (a) had no formal coursework related to sensory processing disorders and A SECRET problem solving strategies; (b) had access to an internet-capable computer; (c) could complete the online foundational instruction for sensory processing and sensory processing disorders from Sensory Processing Disorder University (SPDU); and (d) could complete the A SECRET instructional RLO modules via the Moodle LMS (learning management system) during the designated two-week time frame.

### 2.3. Method of Subject Recruitment

Upon institutional IRB approval, the participants were obtained through purposeful sampling methods at the targeted university. A total of 12 subjects were recruited from the first year MOT class. Of these initial 12, there were eight participants who completed the study. The remaining four withdrew for various reasons (lack of time or semester workload issues).

### 2.4. Design

A case study research design [19] was used to better understand the learners' experience with specific interaction events with the e-learning system, multimedia case scenarios, and researcher-created RLOs. The primary research question addressed in the investigation was what is the level of occupational therapy (OT) students' problem-solving performance for A SECRET after viewing a simulation case study of a child with Sensory Over-Responsiveness (SOR) as measured by a postsimulation selected response assessment?

### 2.5. Procedures Applied to Participants

Once the researcher obtained participants' signed informed consent, they were instructed to complete the sequenced instructional modules with content related to (a) sensory processing, (b) sensory modulation, (c) sensory processing difficulties, and (d) sensory over responsiveness via the SPDU modules. Upon completion of this foundational knowledge, participants began the nine RLOs (strung together) related to A SECRET organized into an instructional module and accessed within a learning management system at the targeted university. Subjects were then presented with a multimedia (video, audio, and text) simulated case scenario followed by a multiple-choice assessment (i.e., a quiz within the LMS) pertaining to each of the seven elements of A SECRET, one question for each element. Within the quiz, participants were required to rank-order (i.e., 1 = appropriate to 6 = inappropriate) a list of six predetermined strategies for each element and provide a rationale/justification for the strategies ranked as 1 and 2 and 5 and 6, considered by the participant to be appropriate (a rating of 1 or 2) and justification for why they chose the ranking of “inappropriate” (a position of 5 or 6). Finally, participants were directed to move to the next element within the assessment. They could move back and forth to different questions within the assessment; however, access to the previously viewed client history or video vignette while completing the assessment was prohibited by design. This was done in order to simulate clinical observation challenges found within existing clinical experiences in pediatric practice.

### 2.6. Description of the Measure

Given the unique nature of the content and delivery of the A SECRET instructional modules and the targeted participants for this study (1st year MOT students) a novel measurement tool was designed and developed as a part of the instructional design process of the overall project in order to capture the student performance and reasoning, specifically, to determine if 1st year MOT students could discriminate between predetermined A SECRET strategies that were categorized as appropriate, adequate, and inappropriate using the content learned from the instruction and contextual information gleaned within a case scenario.

**Figure 2:**
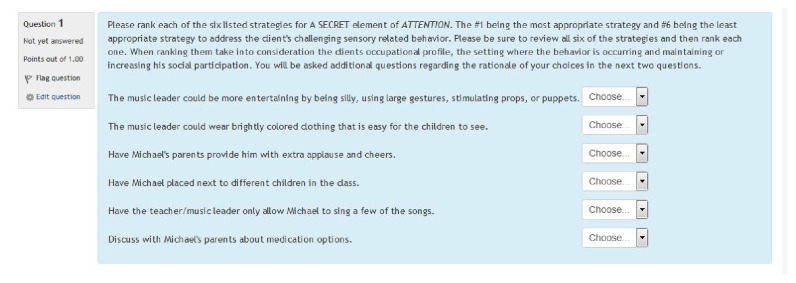
Sample A SECRET selected response question.

**Figure 3:**
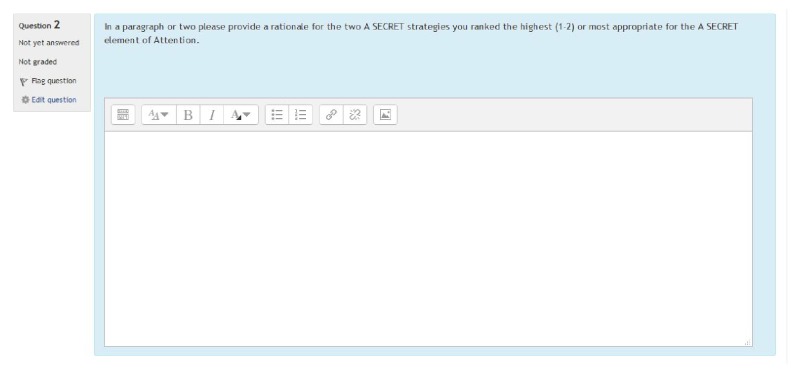
Sample A SECRET rationale of appropriate strategy ratings.

**Figure 4:**
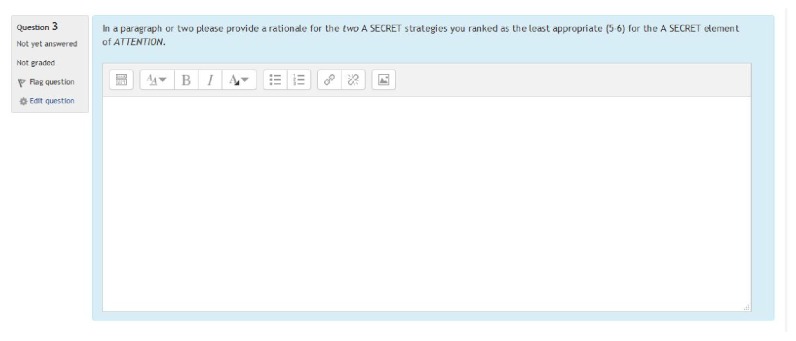
Sample A SECRET rationale of inappropriate strategy ratings.

Fourteen occupational therapy practitioners who had experience with evaluating and treating children with SPD and/or implementing the A SECRET reasoning approach assisted with the process of developing the measure. The measure included a client history/evaluation (diagnostics, evaluation findings, and parental report) and a five-minute video vignette documenting his occupational performance deficits rooted in sensory processing. The initial rounds of strategy generation, included six occupational therapy practitioners who reviewed the case scenario and generated a list of viable strategies for each element of A SECRET. Grammatical revisions and a finalization of the initial strategy list were made to the measure. Eight additional expert reviewers then ranked each of the six strategies for each element of A SECRET. The strategies were grouped into three categories, appropriate, adequate, and inappropriate, based upon a minimum of 80% consensus of the 8 expert reviews. A second review and finalization were conducted to incorporate the ratings and other comments/corrections made by the expert reviewers. Please refer to Figures 2–4 for an example of the measure (presented in a learning management system).

A total of 6 points were possible for each element category on the assessment with a total of 42 points. However, based upon how the assessment was developed, the participant could receive 100% by getting all 6 of the rankings in the correct order. They could receive 67% by getting up to 4 ranked correctly, but they were unable to get 83% with five of the rankings correct because each strategy was assigned a ranking and participants had to provide a different ranking for each strategy; thus, making an error with one strategy actually produced two errors.

During the instructional design process, it was estimated that, generally, a 70% overall on the A SECRET Case Scenario Assessment would constitute a “good” score given the limitations of the ranking system and the novelty of the pilot implementation for this kind of assessment process.

## 3. Findings

All eight participants completed the A SECRET Case Scenario assessment, which required identification and ranking of the two most appropriate strategies for that element (signified by the number 1 or 2); ranking the two strategies that were inappropriate (designated as 5 or 6) and the remaining two categorized as adequate (identified as 3 or 4) from a predetermined list of six strategies for each element. Participants' rankings were compared to the grading key, in which one point was given for each ranking that fell within the correct category of appropriate, inappropriate, or adequate. If the participant ranked a strategy in the wrong order but in the correct category, it was still considered accurate. Descriptive statistics (mean, mode, median, standard deviation, range, and sum) were calculated for each category of the assessment using SPSS 23.0 [20] for the participants' aggregate scores on the selected response portion of the A SECRET case scenario assessment (refer to Table 1).

When looking at the aggregate performance, participants scored the lowest in the category of* environment* (M = 2.37, SD = 0.91) while 100% obtained full credit in the category of* relationships* (M = 6, SD = 0), as depicted in Table 2. The overall research question was to determine if students could discriminate between and correctly rank the appropriate (two strategies) and inappropriate (two strategies) A SECRET strategies, where tracking performance on the neutral strategies was not scored. The cohort of participants attained an overall score of 68% which was 2% shy of the predetermined threshold of 70% established during the design of the measure and instructional intervention.

## 4. Discussion

This is the first study of its kind exploring the effectiveness of an e-learning module to influence knowledge and reasoning of OT students. Previous research that have been conducted within the OT profession has focused on using e-learning technologies to augment level II clinical rotations [21–26], which typically occur at the end of a student's courses of study.

The results from this study indicated the overall participant performance (*n* = 8) on the A SECRET selected-response assessment were two percentage points away from the initial achievement criterion (70%) established by the researcher during the Analyze phase of the premodule instructional design. This indicates a positive finding regarding the instructional influence of the researcher's A SECRET module, given that participants were MOT students in their first year of study who had not completed any courses related to OT interventions (e.g., psychosocial or behavioral dysfunction in adults and/or children). Without implementing the A SECRET case scenario as both a pre- and a postmeasure, it was difficult to determine how much the RLOs could have impacted the participants' knowledge and application of A SECRET.

The participants' specific performances with the* attention* and* relationship* elements exceeded the predetermined threshold and indicate those RLOs were effective in informing them of the content and the process related to these portions of A SECRET. It is further argued the participants' overall performance on the assessment (i.e., approaching the targeted threshold of 70%) adds merit to the effectiveness of the RLOs in teaching the MOT students to logically reason through challenging behaviors related to sensory processing through a simulated format.

Additionally, these participants had taken a course during the second semester in the curriculum that focused on the task (activity) analysis procedure. This may be why they scored well on the* task* subtest on the assessment. However, it is difficult to ascertain why each participant accurately ranked each item on the* relationship* subtest. It may be assumed that the items were too easy or that the items ranked as appropriate versus inappropriate were too obvious to the participants. However, it is also likely the relationship RLO was the most effective at conveying the information through the use of quality multimedia, content, case examples, and instructional design strategies.

The participants' overall mean of correct rankings of strategies was 68% on the selected-response portion of the A SECRET case scenario. This is below the 70% targeted criterion established by the researcher as part of the instructional design process for the A SECRET module. This original threshold was determined due to several factors: (1) the novelty of the instruction; (2) the uniqueness of the content; and (3) the lack of in-depth exposure to the intervention process for specific conditions and populations at this point in the program. The participants were able to reach, or exceed, the 70% criterion for two elements of A SECRET:* attention* and* relationship*. It is difficult to ascertain why the participants performed well with these two elements but not on the other five. The participants scored less than 70% on the latter four elements (*sensation*,* emotional regulation*,* culture*,* environment*, and* task*). The participants scored within two percentage points on* sensation* and* emotional regulation*, which is promising given the novelty of the instruction and the measure. However, of those five, Miller et al. [27] have reported that the element of* culture* is the most difficult to teach and for clients/caregivers to understand.

*Culture* as a part of the A SECRET process was difficult to define, especially in the context of the A SECRET reasoning approach, as it was poorly operationalized. Culture may be interpreted differently by therapists, parents, or caregivers. In future studies, the RLO containing the element* culture* could be enhanced with additional video-based examples incorporating diverse applications of strategies with different sensory problem difficulties or different ages of children.

To date, there is one published study using RLOs within occupational therapy entry-level education [28] and one in OT clinical practice [13]. The findings from the Gee et al. [28] and the Gee et al. [13] investigations indicated the value of RLOs as an adjunctive instructional resource to support student learning. In this current research investigation, given that this was the participants' first exposure to the A SECRET reasoning process, their performance should be considered promising. Furthermore, this is the first study of its kind exploring the use of RLOs to instruct students related to structured, prescriptive reasoning process and, specifically, the A SECRET reasoning approach.

### 4.1. Limitations and Recommendations for Future Research

The nature of the case study approach that was used had inherent limitations, including the inability to generalize, limited opportunity to conduct analysis beyond descriptive statistics, and a narrower focus of questioning and discovery. Therefore, it is recommended that additional research be conducted using the same instructional content and delivery, but with an approach that permits a larger sample size and includes using control and experimental groups.

An increased sample size would allow for the exploration of relationships between the four constructs on the attitudinal survey and/or different demographic profiles of the participants. The above-mentioned design recommendations could be accomplished using MOT students enrolled in their first year from ISU as well as students from larger academic institutions.

Using cohorts of students within the ISU MOT program could result in varying types of opportunities for further research and development. Assessing student performance across the three different cohorts within the ISU MOT program to evaluate their performance with the selected-response case scenario, as well as tracking differences with how each cohort approached the case scenario would provide insight. Performance of participants who took part in the A SECRET module as a stand-alone instructional activity versus those who had the module imbedded within a formal course relevant to the A SECRET process (e.g., pediatric practice courses) could also be examined.

Another possibility would be to determine if the ISU A SECRET module could be implemented within courses that address adults diagnosed with mental illness who also exhibit symptoms related to sensory processing disturbances. A case study could be conducted assessing the attitudes of students regarding the existing content and the difficulty or ease of aligning the content to adult populations within* Psychosocial Dysfunction in Occupation*.

For this study, the A SECRET instructional module was developed to target a variety of learners, although it was tested using 1st year MOT students. The module could be implemented in a different instructional context with caregivers, parents, and teachers of children with sensory processing disturbances. This could be conducted within the context of the Sensory Processing Disorder University to evaluate performance within the instructional content module and then the long-term outcomes (three, six, and 12 months after instruction) in order to assess retention as well as performance in implementing it with their own child in addressing behaviors that are rooted in sensory processing.

### 4.2. Implications for Occupational Therapy Education

The procedures used in this design and development of the RLOs and instructional module in preparation for the study provide examples that may enhance occupational therapy education. The authors used a formal instructional design process and procedure as a mechanism to design and develop the instruction, specifically, the ADDIE model of instructional design [29]. Approaching instructional problems and/or opportunities using a formal approach may ensure that the instruction designed is of higher quality and is meeting the needs of the targeted population [29]. Using simulated case studies, which are paired with specific content, may support the students understanding of content and also the application of some types of clinical reasoning such as procedural and conditional reasoning. Providing instruction that supports structured reasoning early in a curriculum may also support student learning experiences during clinical rotations, as they arrive with specific tools instead of muddied pragmatic approaches often employed by practicing clinicians. Instructional technology such as RLOs sequenced as an instructional module may enhance clinical reasoning strategies such as those employed by the A SECRET reasoning approach to challenging behavior related to sensory processing.

## Figures and Tables

**Table 1:** Descriptive statistics for the ASECRET case scenario assessment.

A SECRET category							
Statistics	Attention	Sensation	Emotion regulation	Culture	Relation	Environment	Task
*N*	8	8	8	8	8	8	8
M	4.12	3	3.37	3.12	6	2.37	4
Mdn	3.5	3	3.5	3.5	6	2	4
Mode	3	2	2	4	6	2	4
Std dev.	1.64	0.92	1.40	1.72	0	0.91	1.51
Range	4	2	4	5	0	3	4
Sum	33	24	27	25	48	19	32

**Table 2:** A SECRET case scenario raw scores.

Participant	Attention total	Sensation total	Emotional regulation total	Culture total	Relationships total	Environment total	Task total	Overall score
01	4	4	3	2	4	2	4	23
02	2	4	2	1	4	1	2	16
03	4	2	3	4	4	1	2	20
04	2	2	2	1	4	1	4	16
05	3	2	3	3	4	2	3	20
06	3	3	3	3	4	0	2	18
07	2	3	2	2	4	1	2	16
08	4	2	4	3	4	3	3	23
Element sum score	*24*	22	22	19	*32*	11	*22*	**152**
% of correct appropriate versus inappropriate strategy rankings	***75%***	68%	68%	60%	***100%***	34%	*68%*	**68%**
